# Endoscopic full-thickness resection versus endoscopic submucosal dissection for colorectal lesions: a systematic review and meta-analysis

**DOI:** 10.3389/fonc.2026.1887590

**Published:** 2026-08-14

**Authors:** Lei Wang, Ting Zhang, Juan Du, Lidan Yang, Wenquan Yi

**Affiliations:** 1 West China Hospital Sichuan University, Meishan Hospital, Meishan, China; 2 Department of Gastroenterology, Meishan People’s Hospital, Meishan, China

**Keywords:** colorectal lesions, endoscopic full-thickness resection, endoscopic submucosal dissection, meta-analysis, systematic review

## Abstract

**Objective:**

To conduct a systematic review and meta-analysis comparing the therapeutic efficacy of endoscopic full-thickness resection (EFTR) and endoscopic submucosal dissection (ESD) for colorectal lesions.

**Methods:**

A comprehensive search of PubMed, Web of science, Cochrane Library and Embase databases until October 1, 2025, supplemented by manual reference screening, identified studies comparing EFTR and ESD. Studies were selected based on predefined inclusion/exclusion criteria, with methodological quality assessed using standardized tools. Meta-analysis was performed using RevMan 5.4, calculating pooled odds ratios (OR) and standardized mean differences (SMD) with 95% confidence intervals.

**Results:**

Five studies involving 570 patients were analyzed. EFTR and ESD showed comparable en bloc resection rates (OR = 2.59, 95%CI 0.38 ∼ 17.77, P = 0.33) and R0 resection rates (OR = 1.73, 95%CI 0.71 ∼ 4.20, P = 0.23). EFTR demonstrated superior safety profiles with significantly lower postoperative perforation risk (OR = 0.13, 95%CI 0.05 ∼ 0.37, P < 0.001), reduced post-polypectomy syndrome incidence (OR = 0.23, 95%CI 0.08 ∼ 0.68, P = 0.008), and shorter procedure time (SMD=-1.97, 95%CI -2.95 ∼ -0.99, P < 0.001). No significant differences were observed in postoperative bleeding (OR = 0.96, 95%CI 0.31 ∼ 2.94, P = 0.94) or lesion recurrence rates (OR = 2.47, 95%CI 0.76 ∼ 8.01, P = 0.13).

**Conclusions:**

In summary, EFTR yields favorable short-term outcomes for selected small or refractory colorectal lesions, particularly non-lifting or recurrent lesions, whereas ESD remains essential for larger superficial colorectal neoplasia requiring en bloc resection.

**Systematic Review Registration:**

https://www.crd.york.ac.uk/PROSPERO, identifier CRD42024534672.

## Introduction

With the heightened utilization of colonoscopy, there has been a notable rise in the detection of colorectal lesions (1, 2). Endoscopic intervention is generally preferred for the management of these lesions due to its minimally invasive characteristics (3). Endoscopic mucosal resection (EMR) is considered effective and safe for the treatment of most small and superficial tumor-like colorectal lesions (4). However, for larger lesions and those with a potential risk of submucosal infiltration, endoscopic submucosal dissection (ESD) is the currently recommended approach (5). Nonetheless, ESD is constrained by a heightened risk of complications and potential failure in achieving en bloc resection, particularly in cases involving non-lifting signs (6), recurrent or incompletely resected lesions, and lesions located in the ileocecal region or in proximity to diverticula (7, 8).

Endoscopic full-thickness resection (EFTR) is an innovative technique for transmural resection of colorectal lesions (9), employing a combination of over-the-scope clip (OTSC) application and cap-assisted snare resection (10). EFTR is usually used as a rescue intervention measure for lesions that are not feasible to treat with EMR/ESD (11). Because it is independent of lesion location or invasion depth and is capable of incising the serosal layer to achieve full-thickness resection (12). EFTR has been proven to be effective in the treatment of non-lifting sign lesions (8), early cancer lesions (13), local residual tumor lesions (8), lesions with deep submucosal invasion (14), and lesions in difficult anatomical sites (15).

At present, several studies have compared the effectiveness and safety of ESD and EFTR in the management of colorectal lesions, yielding varied outcomes (8, 16). Consequently, this study seeks to perform a systematic review and meta-analysis to compare the clinical efficacy and safety of ESD and EFTR in treating colorectal lesions, thereby providing a scientific foundation for selecting appropriate therapeutic strategies.

## Materials and methods

### Search strategy

This study was conducted in accordance with the guidelines of the Preferred Reporting Items for Systematic Reviews and Meta-Analyses (PRISMA-2020) (17). A computerized search was performed in PubMed, Web of science, Cochrane Library and EMbase databases to identify all clinical studies comparing the efficacy of ESD and EFTR for colorectal lesions, from the establishment of the databases to October 1, 2025, the search was restricted to articles published in English. At the same time, the references of the included literature were searched manually to avoid missing possible relevant studies. The search strategy used a combination of MeSH terms and keywords. Specific search terms included: (“Endoscopic full-thickness resection” OR “EFTR” OR “FTRD” OR “ full-thickness resection device”) and (“Endoscopic Submucosal Dissection” OR “ESD”) and (“colorectal “ OR “colonic “ OR “rectal “) and (“lesions” OR “neoplasm” OR “tumour” OR “tumor”). The complete search strategies for all databases are provided in **Supplementary Table 1**.

### Inclusion and exclusion criteria

The inclusion criteria were as follows: (1) the type of study was a control study; (2) the subjects were patients (aged > 18) with endoscopically confirmed colorectal lesions; (3) the EFTR was performed in the experimental group, while the ESD was performed in the control group. The exclusion criteria were: (1) reviews and conference papers; (2) papers with only abstracts in full text and papers that were published repeatedly; (3) papers with incomplete data or papers with specific data that could not be obtained.

### Data extraction

Two reviewers independently screened literatures, extracted data and evaluated the methodological quality of the studies according to the inclusion and exclusion criteria. In case of disagreement, they discussed with each other or reached a third-party ruling. Data extraction included: (1) basic information of the studies, including the title, author, journal name and publication time (year, volume, issue), etc.; (2) type of study design; (3) general information of the studies, including the location of the study, the number of samples, patient age, gender ratio and lesion location and diameter, etc.; (4) histopathological features of lesions: adenoma grade (LGD/HGD), sessile serrated lesion (SSL), intramucosal/T1 carcinoma, submucosal invasion depth (SM1/SM2-3), proportion of non-lifting fibrotic lesions and post-EMR residual/recurrent scar lesions; (5) outcome indicators, including procedure time, resected specimen size, postoperative perforation rate, bleeding rate, en bloc resection rate, R0 resection rate and recurrence rate. If the data were incomplete or missing information, the corresponding author was contacted by telephone or email to obtain.

### Outcome definition

Postoperative perforation was defined as new-onset peritoneal irritation signs plus confirmation of free intraperitoneal air on imaging, applicable to both EFTR (encompassing failed closure or delayed leakage) and ESD. Post-polypectomy syndrome required clinical symptoms (pain, fever, leukocytosis) and CT findings of wall thickening without free air, distinct from true perforation. R0 resection required histologically negative margins ≥ 1mm. Recurrence mandated endoscopic and histopathological confirmation of neoplasia at the site. Bleeding was defined as clinically significant hemorrhage necessitating endoscopic intervention, blood transfusion, or associated with a hemoglobin drop ≥ 2g/dL. Follow-up duration and surveillance protocols were extracted verbatim, with most studies adhering to 6- and 12-month colonoscopy schedules.

### Risk-of-bias assessment

The included RCTs were evaluated for literature quality using the Risk of Bias 2 (RoB 2) tool (18) recommended in the Cochrane Handbook of Systematic Reviews of Interventions. The literature was rated as “low risk”, “some concern”, or “high risk” based on the strength of evidence. For the included non-RCT, the Risk Of Bias In Non-randomized Studies - of Interventions (ROBINS-I) (19) was used to assess the risk of bias across seven domains: confounding bias, selection bias, classification bias, information bias, missing data bias, reporting bias, and other biases. For each domain, the risk of bias was categorized as “low risk,“ “moderate risk, “ or “high risk”.

### Statistical methods

RevMan 5.4 software provided by Cochrane was used for Meta-analysis. Standard mean difference (SMD) and 95%CI were used for effect analysis of continuous variables; odds ratios (OR) and 95%CI were used for effect analysis of categorical variables. The χ2 test and I^2^ statistic were utilized to assess heterogeneity. For studies reporting medians and interquartile ranges (IQR) rather than means and standard deviations (SD), we estimated means and SDs using the method proposed by Wan et al. (20) for meta-analysis of continuous outcomes with skewed distributions.

If the P value was ≥ 0.1 and I^2^ was < 50%, it indicated no significant heterogeneity among the studies. Conversely, if the P value was < 0.1 and I^2^ was ≥ 50%, it suggested significant heterogeneity among the studies. The random effects model was used to combine effect sizes to more conservatively estimate the overall effect and its 95% confidence interval. Sensitivity analysis was conducted by eliminating individual studies one by one to test the stability of the combined results. Egger’s linear regression test and the construction of funnel plots were used to assess potential publication bias. A significance level of α=0.05 was set for meta-analysis, with results considered statistically significant if the P value was < 0.05.

## Result

### Literature search results

**Figure 1:**
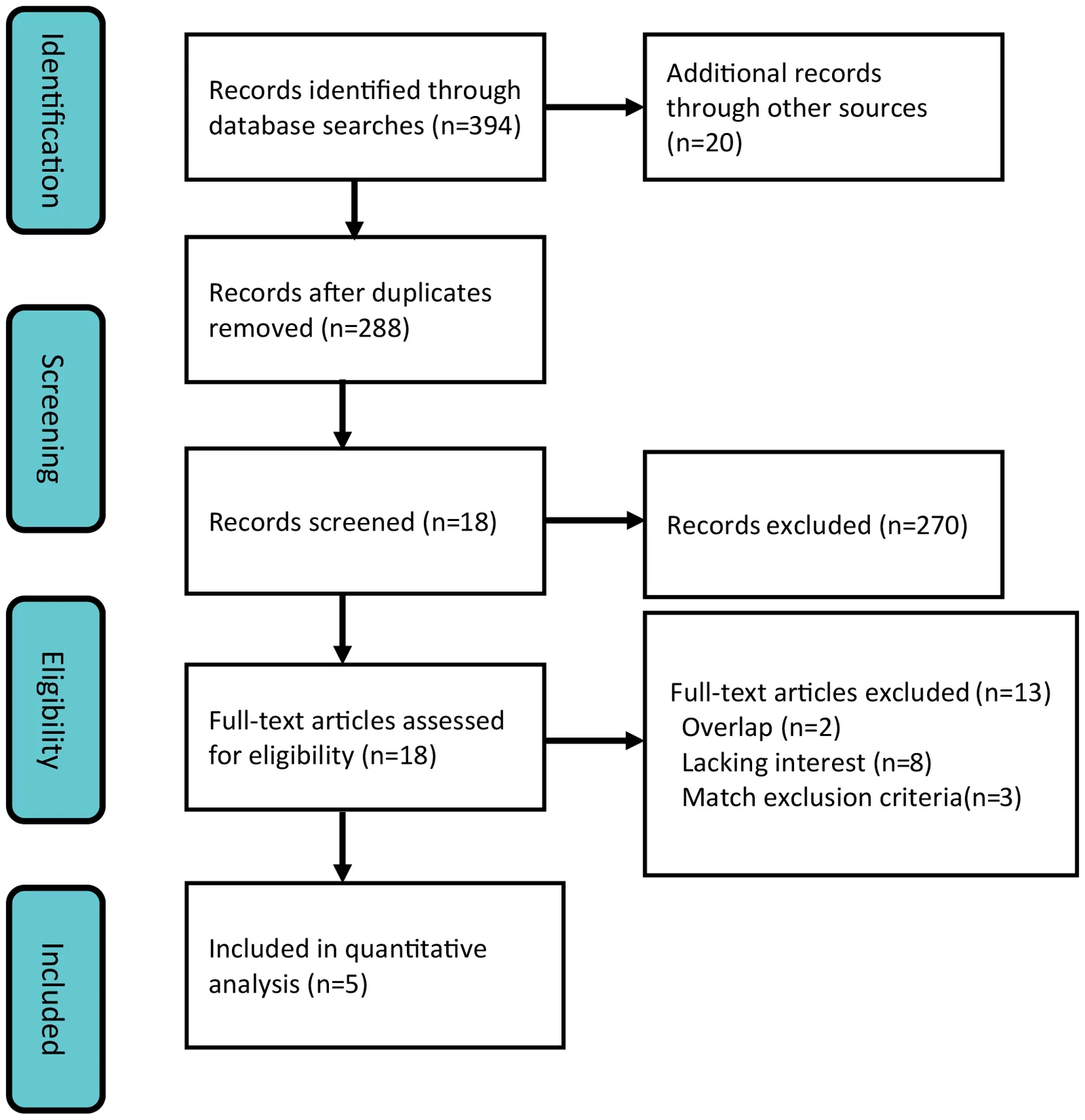
Flow chart for the literature search.

According to the predefined search strategy, a total of 414 potentially relevant records were initially identified. After removing 126 duplicate records, 288 unique records underwent title and abstract screening, during which 270 irrelevant studies were excluded. Subsequently, 18 full-text articles were retrieved and evaluated for eligibility. Ultimately, 5 studies met the inclusion criteria, comprising 1 RCT (16) and 4 non-RCT (8, 16, 21, 22) studies. The complete screening workflow is illustrated in **Figure 1**.

### Description of included studies

The five included studies comprised a total of 570 patients, with 242 in the EFTR group and 328 in the ESD group. In three of these studies (8, 21, 22), the tumor size in the ESD group was significantly larger than in the EFTR group. The basic characteristics of the included studies are summarized in **Table 1**, while the clinical outcome indicators are detailed in **Supplementary Table 2**.

**Table 1:** Study characteristics of publications included in the systematic review and meta-analysis.

Author	Year	Study design	Country	Patients (n)		Age (year)		Lesion location		Tumor size (cm)	
				EFTR	ESD	EFTR	ESD	EFTR	ESD	EFTR	ESD
Bisogni	2020	Single-center retrospective	Italy	27	13	69.8±13.3	74.9 ± 7.8	27^a^	13	1.93 ± 1.06	4.91 ± 1.75
Falt	2021	Single-center retrospective	Czech Republic	52	50	69.1 ± 10.3	66.3 ± 10.5	7/15/8/10/12^b^	32/13/3/0/2	1.56 ± 0.71	2.17 ± 0.42
Yzet	2023	Multicenter retrospective	French	98	177	71.0 ± 2.7	69.5 ± 3.0	22/26/15/22/13	54/35/12/45/31	(0.3–3)	3.5 (0.5–14)
Andrisani	2023	multicenter P-RCT	Italy	45	45	69.1 ± 9.2	68.2 ± 10.8	11/8/14/8/4	9/13/11/11/1	NA	NA
Barbaro	2024	Multicenter retrospective	Italy/Japan	20	43	67.3 ± 14.3	66.8 ± 13.2	7/4/9^c^	11/21/11	1.7 ± 0.77	1.67 ± 0.84

^a^ Lesion located in rectum.

^b^ Lesion located in rectum/left colon/transverse colon/right colon/cecum.

^c^ Lesion located in left colon/transverse colon/right colon.

EFTR, endoscopic full-thickness resection; ESD, endoscopic submucosal dissection; NA, not available; P-RCT, prospective randomized study.

We extracted histopathological data from all five included studies and summarized group-level lesion features in **Supplementary Table 3**. ESD cohorts carried larger lesions with more SM2/SM3 deep submucosal invasion in Bisogni et al. (21), Falt et al. (22) and Yzet et al. (8) research. Falt et al. (22) reported far more fibrotic recurrent lesions in the EFTR arm, while Bisogni et al. (21) and Yzet et al. solely recruited post-EMR recurrent lesions. Notably, the Bisogni et al. (21) cohort had a markedly higher T1 carcinoma proportion in the ESD group.

There were certain differences in the follow-up duration and monitoring protocols in the included studies. The median follow-up period ranged from 12 to 24 months, and the follow-up duration varied from 6 to 48 months. Andrisani et al. (7) and Bisogni et al. (21) used shorter monitoring windows (6–24 months), but Yzet et al. (8) implemented the most stringent protocol, conducting evaluations at 1, 3, 6, and 12 months after surgery, followed by one evaluation per year thereafter. Loss to follow-up was minimal across all cohorts (<2% per arm), as detailed in **Supplementary Table 4**.

### Risks of bias

One RCT (16) included in this study was assessed as low risk of bias using the RoB 2.0 (**Supplementary Figure 1**), with adequate randomization and allocation concealment, consistent intervention implementation, complete outcome data, and no selective reporting. The ROBINS-I assessment indicated a moderate risk of bias for the four retrospective studies, primarily driven by confounding such as baseline imbalance in lesion size and potential selection bias (**Supplementary Table 5**).

### Resection outcome of EFTR and ESD

**Figure 2:**
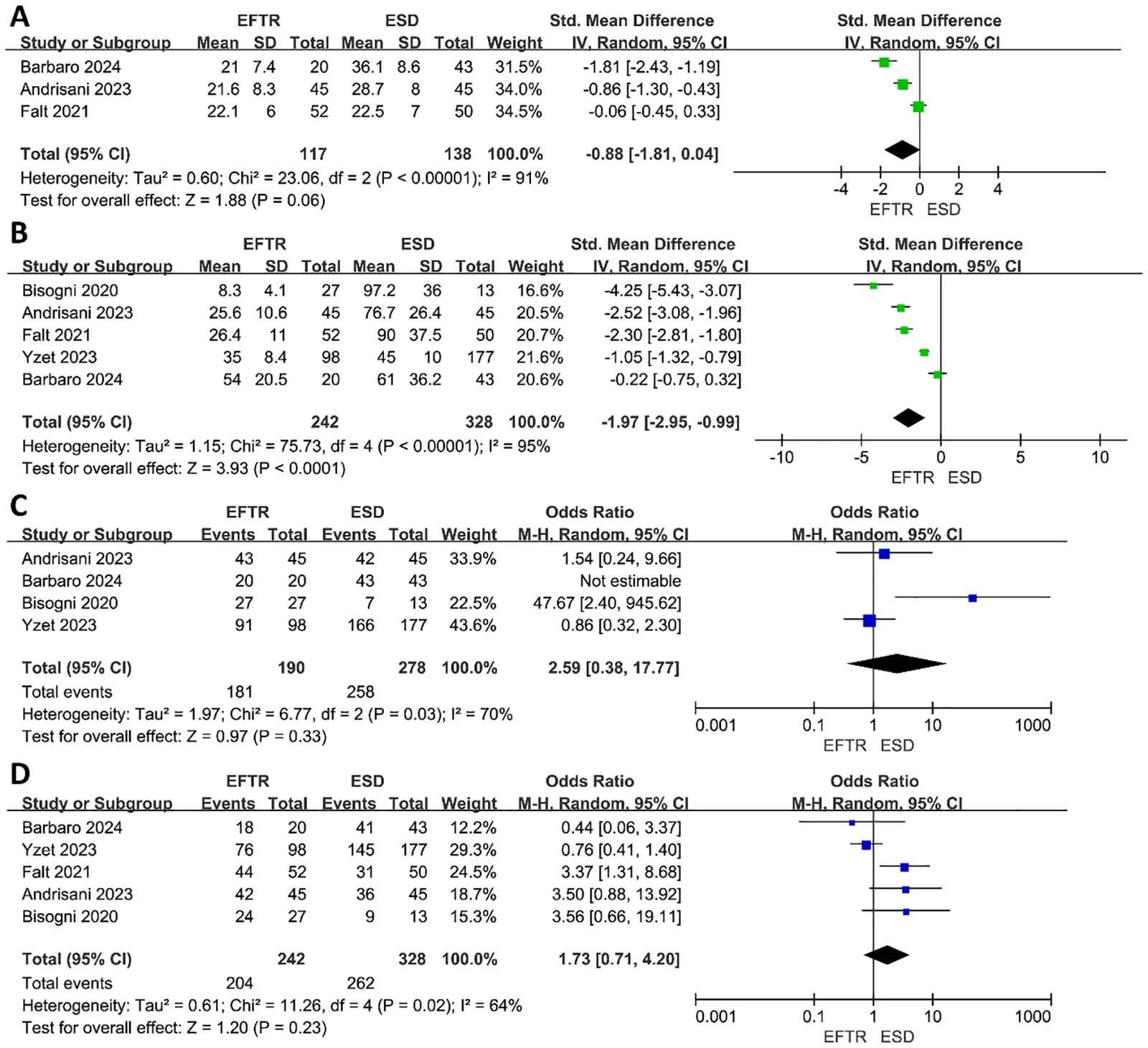
Meta-analysis for resection outcome: **(A)** specimen diameter; **(B)** procedure time; **(C)** en bloc resection rates; **(D)** R0 resection rates.

Three studies (7, 16, 22) reported the diameter of the specimen. Heterogeneity tests indicated that there was significant heterogeneity among studies (I^2^ = 91%). The meta-analysis results indicated no statistically significant difference in the diameter of resected specimens between EFTR and ESD (SMD=-0.88, 95%CI -1.81 ∼ 0.04, P = 0.06), as shown in **Figure 2A**.

Five studies (7, 8, 16, 21, 22) provided data on procedure time. Heterogeneity tests indicated that there was significant heterogeneity among studies (I^2^ = 95%). The meta-analysis results of the meta-analysis showed that the procedure time of the EFTR group was significantly lower than that of the ESD group (SMD=-1.97, 95%CI -2.95 – -0.99, P < 0.001), as shown in **Figure 2B**.

Four studies (7, 8, 16, 21) reported en bloc resection rates, with 95.3% (181/190) achieved in the EFTR group versus 92.8% (258/278) in the ESD group. Heterogeneity tests indicated that there was significant heterogeneity among studies (I^2^ = 70%). The meta-analysis results indicated no statistically significant difference in en bloc resection rates between EFTR and ESD (OR = 2.59, 95%CI 0.38 ∼ 17.77, P = 0.33), as shown in **Figure 2C**.

Five studies (7, 8, 16, 21, 22) provided data on R0 resection rates, with 84.3% (204/242) achieved in the EFTR group versus 79.9% (262/328) in the ESD group. Heterogeneity tests indicated that there was significant heterogeneity among studies (I^2^ = 70%). The meta-analysis results revealed no significant difference in R0 resection rates between EFTR and ESD (OR = 1.73, 95%CI 0.71 ∼ 4.20, P = 0.23), as shown in **Figure 2D**.

### Complications of EFTR and ESD

**Figure 3:**
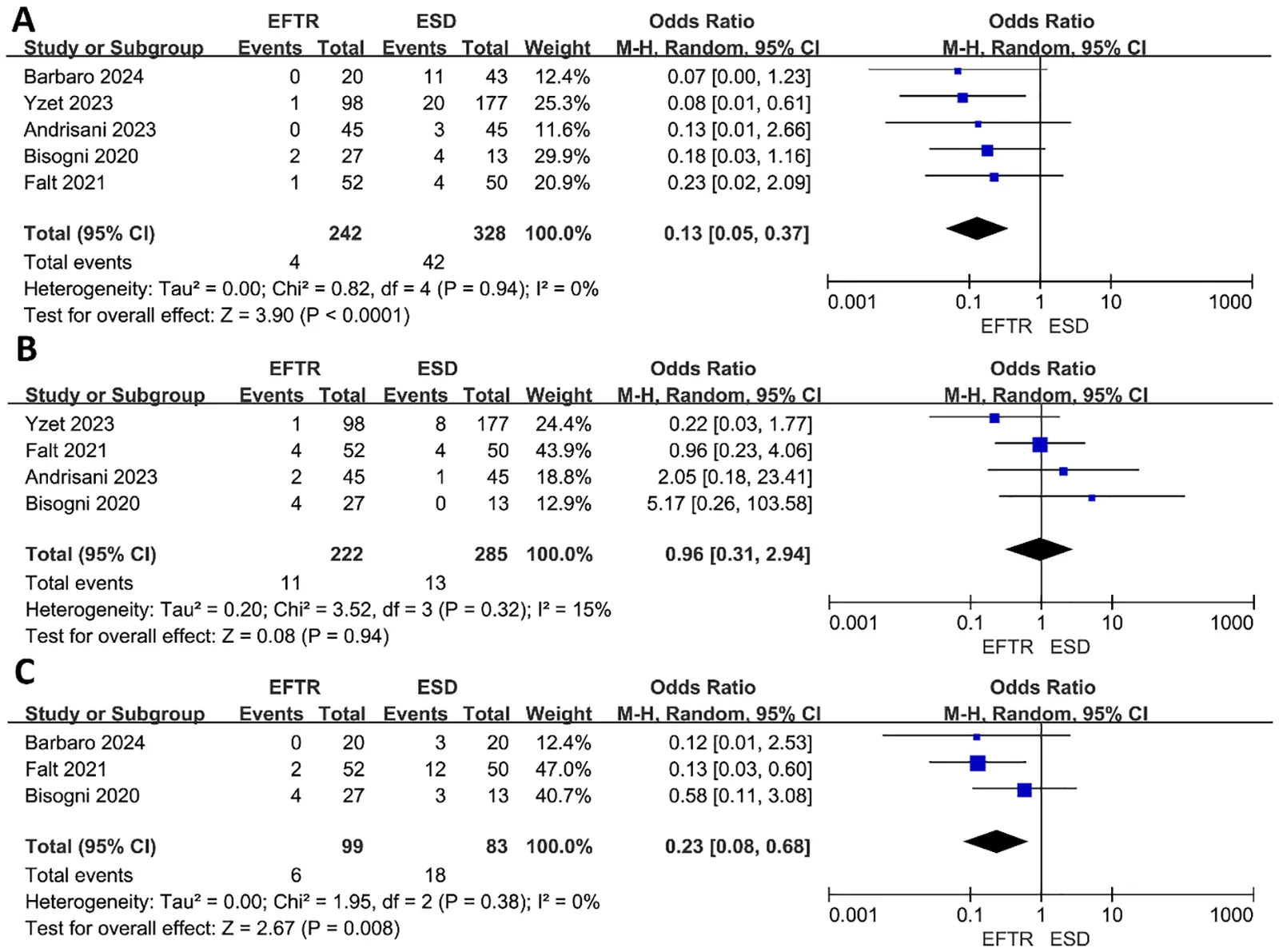
Meta-analysis for complications: **(A)** postoperative perforation; **(B)** postoperative bleeding; **(C)** post-polypectomy syndrome.

All five studies (7, 8, 16, 21, 22) documented the occurrence of postoperative perforation. Among the EFTR group, 4 out of 242 patients experienced postoperative perforation; whereas in the ESD group, 42 out of 328 patients had postoperative perforation. Heterogeneity tests indicated that there was low heterogeneity among studies (I^2^ = 0%). The results of the meta-analysis indicated that the incidence of postoperative perforation was significantly lower in the EFTR group compared to the ESD group (OR = 0.13, 95%CI 0.05 ∼ 0.37, P < 0.001), as shown in **Figure 3A**.

Four studies (7, 8, 21, 22) observed the occurrence of postoperative bleeding. In the EFTR group, 11 out of 222 patients experienced postoperative bleeding, while in the ESD group, 13 out of 285 patients had postoperative bleeding. Heterogeneity tests indicated that there was low heterogeneity among studies (I^2^ = 15%). The results indicated no significant difference in the incidence of postoperative bleeding between the EFTR and ESD groups (OR = 0.96, 95%CI 0.31 ∼ 2.94, P = 0.94), as shown in **Figure 3B**.

Three studies (16, 21, 22) investigated the occurrence of post-polypectomy syndrome. Heterogeneity tests indicated that there was low heterogeneity among studies (I^2^ = 0%). The results demonstrated a significantly lower incidence of post-polypectomy syndrome in the EFTR group compared to the ESD group (OR = 0.23, 95%CI 0.08 ∼ 0.68, P = 0.008), as shown in **Figure 3C**.

### Hospital stay and recurrence

**Figure 4:**
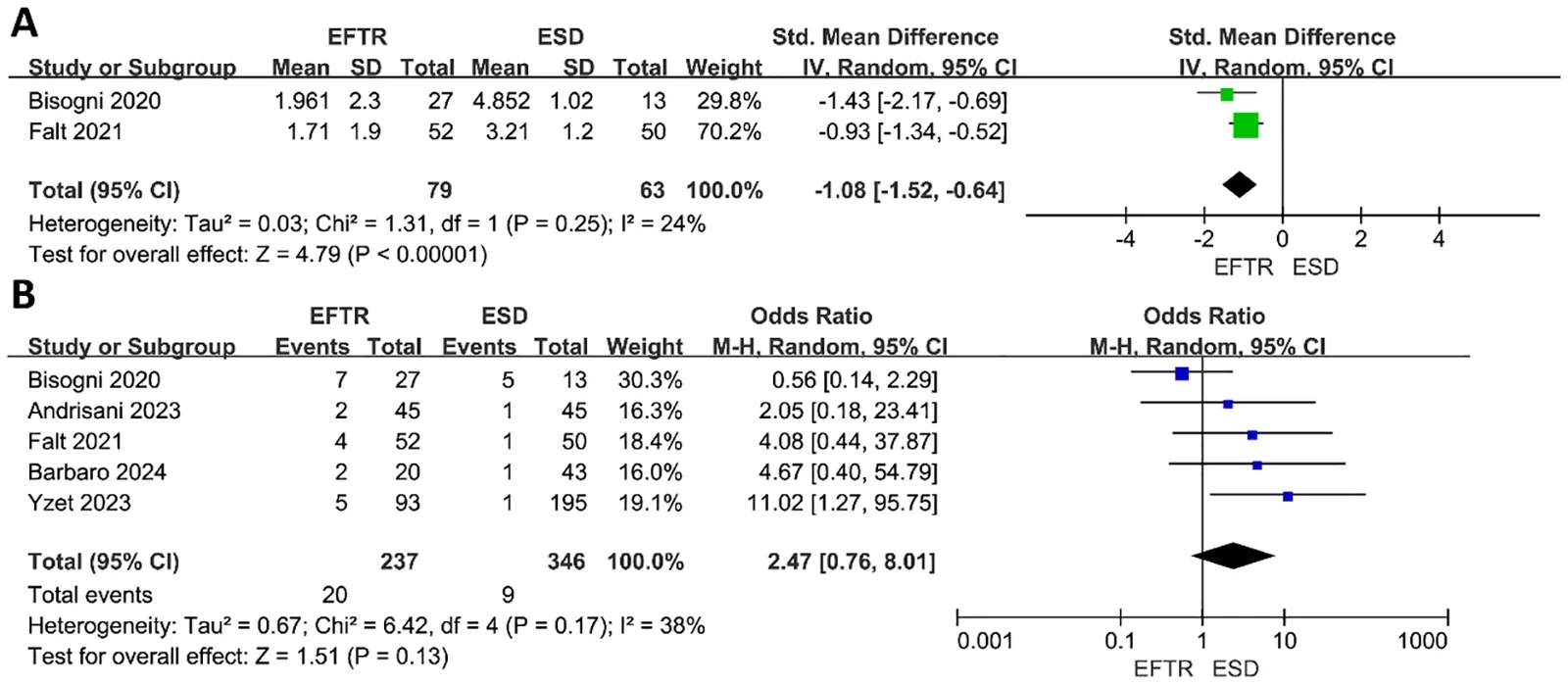
Meta-analysis for hospital stay **(A)** and postoperative recurrence **(B)**.

Two studies (21, 22) examined the hospital stay data. A meta-analysis using a random-effect model revealed that the length of stay in the EFTR group was significantly shorter than that in the ESD group (SMD=-1.08, 95%CI -1.52 ∼ -0.64, P< 0.001), as shown in **Figure 4A**. Additionally, five studies (7, 8, 16, 21, 22) provided data on postoperative recurrence, and a meta-analysis using a random-effect model indicated no significant difference in postoperative recurrence rates between the EFTR and ESD groups (OR = 2.47, 95%CI 0.76 ∼ 8.01, P = 0.13), as shown in **Figure 4B**.

### Sensitivity analysis and publication bias

Sensitivity analysis was performed using the one-by-one exclusion method to evaluate the impact of individual studies on the pooled results. The result[8]s showed that when the study by Yzet et al. (8) was excluded, the pooled effect size of R0 resection rate changed from 1.73 to 2.67, leading to a directional change in the conclusion; when the study by Falt et al. (22) was excluded, the meta-analysis results for diameter of the specimen and the occurrence of post-polypectomy syndrome underwent directional changes, indicating poor robustness of the meta-analysis results for these three outcome measures. We attempted to assess publication bias by plotting a funnel plot (**Supplementary Figure 2**); however, due to the number of included studies being less than 10, standard statistical tests such as Egger’s test had insufficient statistical power, precluding reliable conclusions.

## Discussion

This systematic review and meta-analysis compared the therapeutic efficacy and safety of EFTR and ESD for colorectal lesions, synthesizing data from five studies comprising 570 patients. Our results demonstrate that EFTR yields comparable en bloc (OR = 2.59, 95%CI 0.38~17.77, *P* = 0.33) and R0 resection rates (OR = 1.73, 95%CI 0.71~4.20, *P* = 0.23) relative to ESD. However, EFTR was associated with a significantly shorter procedure time (SMD=-1.97, 95%CI -2.95~-0.99, *P* < 0.001) and a superior safety profile, characterized by markedly lower risks of postoperative perforation (OR = 0.13, 95%CI 0.05~0.37, *P* < 0.001) and post-polypectomy syndrome (OR = 0.23, 95%CI 0.08~0.68, *P* = 0.008).

EMR is primarily suitable for intramucosal lesions with shallow resection depth, but often results in incomplete resection of colorectal lesions (23). The reported R0 resection rate of EMR ranges from 40% to 75% in the literature (4, 24). EFTR and ESD are advanced endoscopic resection techniques for colorectal lesions (25), mainly employed in cases with a risk of submucosal invasion, non-lifting signs caused by submucosal fibrosis, recurrence after previous endoscopic treatment, or periappendiceal lesions (26). The main procedures of ESD include identification and marking of lesion margin, submucosal injection, circumferential incision, and submucosal dissection (27). Compared with traditional EMR, the advantage of ESD is that en bloc resection can be performed on lesions with diameters > 20mm (28), avoiding fragmented resections and subsequent local recurrence. After en bloc resection of the lesion, histopathological analysis can be performed to determine whether it is R0 resection (29). Regardless of the size and location of colorectal lesions, ESD can be performed (28). However, the above advantages come at the cost of increased risks of perforation and bleeding and longer procedure time (30). ESD is a highly difficult operation with a flat learning curve (31). Operators need strict training to minimize complications such as perforation and bleeding (32).

EFTR represents an innovative technique employing specialized equipment, predominantly facilitated by an over-the-scope full-thickness resection device, which enables traction, suturing, closure, and resection (33). EFTR requires less training, significantly shortens surgery time, and may have a lower risk of complications (34). Currently, several studies have been published comparing EFTR and ESD for the treatment of colorectal tumor lesions, necessitating a systematic review and meta-analysis to assess their efficacy and safety. Our meta-analysis results indicate that both EFTR and ESD demonstrate favorable resection effects on colorectal lesions, with no significant difference in en bloc resection rates (OR = 2.59, 95%CI 0.38~17.77, P = 0.33) and R0 resection rates (OR = 1.73, 95%CI 0.71~4.20, P = 0.23). However, this pooled result must be interpreted with caution due to histopathological imbalance across study arms. In our meta-analysis, it was observed that the diameter of colorectal lesions in the EFTR group was significantly smaller than that in the ESD group in three studies (8, 21, 22). EFTR is typically indicated for lesions requiring full-thickness resection, such as those with non-lifting signs due to submucosal fibrosis, post-EMR recurrence, or location in anatomically challenging sites, rather than only for lesions with confirmed full-wall invasion (35). A primary limitation of EFTR is its restricted resection range, generally less than 30 mm, which may be associated with the diameter of the transparent cap (22, 36). According to a single-arm meta-analysis by Dolan et al. (37), EFTR demonstrates safety and efficacy for colorectal lesions measuring less than 20 mm. However, for lesions exceeding 20 mm, the incidence of surgery-related adverse events increases significantly. Nevertheless, EFTR’s ability to directly resect the entire tissue layer allows for precise and complete lesion removal in selected cases.

ESD excels in the management of large mucosal and submucosal lesions (38). By enabling the stepwise dissection of lesion tissue, ESD achieves high resection rates even for lesions with substantial diameters (39). While lesions in the EFTR group were smaller, the inherent procedural characteristics of EFTR confer high efficiency for excising smaller lesions (34). Conversely, despite the larger lesion size in the ESD group, the technical advantages of ESD ensure effective resection of large lesions. This likely explains the absence of significant differences in the en bloc resection rate and R0 resection rate observed between the two groups, despite the disparity in lesion diameter. Furthermore, previous studies have demonstrated that lesion size is not the sole determinant of therapeutic efficacy in endoscopic resection (40). Factors including lesion location, depth of invasion, and histological type also exert a significant impact on resection outcomes (41). While the studies included in this meta-analysis did not perform detailed stratified analyses based on these specific factors, it can be reasonably inferred that, in the overall study population, the combined effects of these confounding variables may have counterbalanced the potential influence of lesion diameter differences on resection efficiency.

It is a well-known fact that the procedure time of colorectal ESD is relatively long (42), and the long procedure time is closely related to the occurrence of postoperative complications. Furthermore, even operators with extensive professional expertise in ESD may encounter challenges (43), such as the presence of high fibrosis in the lesion may still result in a lower en bloc resection rate and a higher perforation rate (44). However, once the lesion is successfully located with the assistance of FTRD instrument, EFTR can complete the resection very quickly (45, 46), bypassing the fine submucosal dissection stage of ESD, which greatly improves the procedure time and overall resection rate (7). Additionally, the use of over-the-scope (OTS) clip effectively controls postoperative perforation rates (47).

Our meta-analysis also suggests that EFTR exhibits significantly shorter procedure times and lower incidence of postoperative perforations compared to ESD.​ However, it is important to note that data regarding hospital stay were available from only two of the five included studies (Bisogni et al. (21) and Falt et al. (22)). While the pooled analysis of these limited datasets suggested a potential reduction in hospitalization duration with EFTR, this finding should be interpreted cautiously due to the small number of contributing studies and considerable clinical heterogeneity.​ The clinical implications of these findings warrant careful consideration. While EFTR’s reduced complication profile is encouraging, its utility remains confined to smaller lesions due to device limitations (22, 36) (e.g., cap diameter). Conversely, ESD’s ability to manage larger lesions with comparable R0 rates suggests it retains a critical role in complex cases; at the same time, for large lesions that require ESD treatment, a longer hospital stay is a reasonable choice to address potential complications of the complex resection.

We observed that EFTR demonstrates favorable safety profiles in the management of rectal lesions. Nevertheless, it is crucial to clarify that this finding does not establish EFTR as a preferred alternative to EMR or ESD for unselected lesions. To improve the clinical applicability of our meta-analysis, it is essential to delineate the current treatment hierarchy of endoscopic techniques for colorectal lesions. In routine clinical practice, EMR or cold snare polypectomy (CSP) remains the first-line approach for small-diameter elevated colorectal lesions, given their ease of implementation, cost-effectiveness, and well-documented safety. For larger or morphologically complex lesions—such as laterally spreading tumors, non-elevated lesions, or lesions with submucosal fibrosis—ESD is more recommended. Despite its technical complexity and prolonged learning curve, ESD enables en bloc resection and ensures adequate resection margins. EFTR may be primarily indicated in complex scenarios where standard endoscopic procedures are unfeasible or have failed. These scenarios include non-resectable lesions attributed to fibrosis or recurrent disease, lesions located in anatomically challenging sites like the appendiceal orifice and diverticula, and cases requiring full-thickness resection to achieve complete tumor clearance.

This meta-analysis has several key limitations. Paramount among these is the fundamental indication mismatch: EFTR was predominantly applied to smaller refractory lesions such as recurrent or scarred lesions, constrained by device cap diameter ≤ 30 mm, whereas ESD addressed larger lesions. This baseline imbalance likely inflates EFTR’s perceived safety advantage and obscures true efficacy differences. Substantial heterogeneity across outcomes, particularly procedure time (I^2^ = 95%), further limits the precision of pooled estimates, precluding definitive inferences for strictly matched populations. Additionally, the inclusion of only five studies (n=570) restricted quantitative assessment of publication bias, while inconsistent follow-up durations ranging 6–48 months and variable surveillance protocols compromise the reliability of recurrence data. Notably, the predominance of retrospective designs (four of five studies) and the lack of granular data stratified by histopathology including adenoma, early cancer, scar, or residual neoplasia resulted in low overall certainty of evidence. Consequently, these findings should be interpreted as hypothesis-generating rather than conclusive. Future prospective, stratified trials are essential to define the comparative roles of EFTR and ESD across specific lesion subtypes.

## Conclusions

In conclusion, EFTR may provide favorable short-term outcomes for selected small or refractory colorectal lesions, particularly non-lifting or recurrent lesions, whereas ESD remains essential for larger superficial colorectal neoplasia requiring en bloc resection. Current comparative evidence is limited by selection bias, small sample size, and substantial clinical and statistical heterogeneity; therefore, prospective stratified studies are required to definitively establish the comparative efficacy and safety profiles of these advanced endoscopic techniques.

## Data Availability

The original contributions presented in the study are included in the article/**Supplementary Material**. Further inquiries can be directed to the corresponding author.
