# Supplementary material for: Endoscopic full-thickness resection versus endoscopic submucosal dissection for colorectal lesions: a systematic review and meta-analysis
Source: Front Oncol. 2026 Aug 14;16:1887590. doi: 10.3389/fonc.2026.1887590 (PMC13521782; doi:10.3389/fonc.2026.1887590)
Supplement: Supplementary file 1 [file DataSheet1.docx]

**Endoscopic full-thickness resection versus** **endoscopic submucosal dissection for colorectal lesions: a systematic review and meta-analysis**

Supplementary Table S1. Detailed search strategies for each database (search date: October 1, 2025)

Supplementary Table S2. Clinical outcomes of EFTR versus ESD in included studies

Supplementary Table S3. Summary of Histopathological and Lesion Characteristics

Supplementary Table S4. Summary of follow-up characteristics across included studies

Supplementary Table S5. Risk of bias for the included the four non-RCTs, based on the ROBINS-I tool

Supplementary Figure S1 Risk of bias for the included the one RCT, based on the RoB 2 tool.

Supplementary Figure S2. Funnel plots of the resection results of EFTR and ESD. (A) Specimen diameter funnel plot; (B) Procedure time funnel plot; (C) En bloc resection rate funnel plot; (D) R0 resection rate funnel plot.

Supplementary Table S1. Detailed search strategies for each database (search date: October 1, 2025)

| Database | Complete search strategy |
| --- | --- |
| PubMed | #1 ("Endoscopic Full-Thickness Resection"[tiab] OR "EFTR"[tiab] OR "Full-Thickness Resection Device"[tiab] OR "FTRD"[tiab] OR "Over-the-Scope Clip"[tiab] OR "OTSC"[tiab] OR "Cap-Assisted Full-Thickness Resection"[tiab] OR "Endoscopic Transmural Resection"[tiab]) #2 ("Endoscopic Submucosal Dissection"[tiab] OR "ESD"[tiab] OR "Endoscopic Submucosal Resection"[tiab]) #3 ("Colorectal Neoplasms/surgery"[Mesh] OR "Colonic Diseases/surgery"[Mesh] OR "Rectal Diseases/surgery"[Mesh] OR "colorectal"[tiab] OR "colonic"[tiab] OR "rectal"[tiab] OR "colon"[tiab] OR "rectum"[tiab] OR "bowel"[tiab] OR "colorectal neoplasia"[tiab]) #4 ("neoplasm*"[tiab] OR "tumor*"[tiab] OR "tumour*"[tiab] OR "lesion*"[tiab] OR "polyp*"[tiab] OR "adenoma*"[tiab] OR "carcinoma*"[tiab] OR "scar recurrence"[tiab]) #5 ("Comparative Study"[pt] OR "Controlled Clinical Trial"[pt] OR "Randomized Controlled Trial"[pt] OR "Observational Study"[pt] OR "cohort stud*"[tiab] OR "case-control stud*"[tiab] OR "comparative stud*"[tiab]) #6 #1 AND #2 AND #3 AND #4 AND #5 |
| Web of Science | TS=(("endoscopic full-thickness resection" OR EFTR OR "full-thickness resection device" OR FTRD OR "over-the-scope clip" OR OTSC OR "cap-assisted full-thickness resection" OR "endoscopic transmural resection") AND ("endoscopic submucosal dissection" OR ESD OR "endoscopic submucosal resection") AND (colorectal OR colonic OR rectal OR colon OR rectum OR bowel OR "colorectal neoplasia") AND (neoplasm* OR tumor* OR tumour* OR lesion* OR polyp* OR adenoma* OR carcinoma* OR "scar recurrence") AND ("comparative study" OR "controlled clinical trial" OR "randomized controlled trial" OR "observational study" OR "cohort study" OR "case-control study")) Language: English; Document types: Article, Clinical Trial, Comparative Study, Controlled Clinical Trial, Randomized Controlled Trial, Observational Study |
| Embase | #1 'endoscopic full thickness resection'/exp OR 'endoscopic full-thickness resection':ti,ab OR EFTR:ti,ab OR 'full-thickness resection device':ti,ab OR FTRD:ti,ab OR 'over-the-scope clip':ti,ab OR OTSC:ti,ab OR 'cap-assisted full-thickness resection':ti,ab OR 'endoscopic transmural resection':ti,ab #2 'endoscopic submucosal dissection'/exp OR 'endoscopic submucosal dissection':ti,ab OR ESD:ti,ab OR 'endoscopic submucosal resection':ti,ab #3 'colorectal tumor'/exp OR 'colonic disease'/exp OR 'rectal disease'/exp OR 'colorectal neoplasia'/exp OR colorectal:ti,ab OR colonic:ti,ab OR rectal:ti,ab OR colon:ti,ab OR rectum:ti,ab OR bowel:ti,ab #4 neoplasm*:ti,ab OR tumor*:ti,ab OR tumour*:ti,ab OR lesion*:ti,ab OR polyp*:ti,ab OR adenoma*:ti,ab OR carcinoma*:ti,ab OR 'scar recurrence':ti,ab #5 'comparative study'/exp OR 'controlled clinical trial'/exp OR 'randomized controlled trial'/exp OR 'observational study'/exp OR 'cohort study':ti,ab OR 'case-control study':ti,ab #6 #1 AND #2 AND #3 AND #4 AND #5 #7 Limit #6 to English language, human, article |
| Cochrane | #1 MeSH descriptor: [Endoscopic Full-Thickness Resection] explode all trees OR EFTR OR "full-thickness resection device" OR FTRD OR "over-the-scope clip" OR OTSC OR "cap-assisted full-thickness resection" OR "endoscopic transmural resection" #2 MeSH descriptor: [Endoscopic Submucosal Dissection] explode all trees OR ESD OR "endoscopic submucosal resection" #3 MeSH descriptor: [Colorectal Neoplasms] explode all trees OR MeSH descriptor: [Colonic Diseases] explode all trees OR MeSH descriptor: [Rectal Diseases] explode all trees OR colorectal OR colonic OR rectal OR colon OR rectum OR bowel OR "colorectal neoplasia" #4 neoplasm* OR tumor* OR tumour* OR lesion* OR polyp* OR adenoma* OR carcinoma* OR "scar recurrence" #5 "comparative study" OR "controlled clinical trial" OR "randomized controlled trial" OR "observational study" OR "cohort study" OR "case-control study" #6 #1 AND #2 AND #3 AND #4 AND #5 |

| Author (Year) | Group | n | Specimen diameter (mm) | Procedure time (min) | En bloc resection* | R0 resection* | postoperative perforation* | Bleeding* | Recurrence* | PPS* | Hospital stay (days) |
| --- | --- | --- | --- | --- | --- | --- | --- | --- | --- | --- | --- |
| Bisogni (2020) | EFTR | 27 | NA | 8.33 ± 4.12 | 27/27 | 24/27 | 2/27 | 4/27 | 7//27 | 4/27 | 1.96 ± 1.02 |
|  | ESD | 32 | NA | 97.15 ± 35.95 | 7/32 | 9/32 | 4/32 | 0/32 | 5/32 | 3/32 | 4.85 ± 2.30 |
| Falt (2021) | EFTR | 58 | 22.1 ± 6.0 | 26.4 ± 11.0 | NA | 44/58 | 1/58 | 4/58 | 4/58 | 2/58 | 1.2 ± 0.5 |
|  | ESD | 55 | 25.2 ± 7.0 | 90–240 (median 135) | NA | 31/55 | 4/55 | 4/55 | 1/55 | 12/55 | 3.2 ± 1.9 |
| Yzet (2023) | EFTR | 98 | NA | 21.3–54.8 (median 35.0) | 91/98 | 76/98 | 1/98 | 1/98 | NA | NA | NA |
|  | ESD | 169 | NA | 30.0–75.0 (median 45.0) | 166/169 | 145/169 | 20/169 | 8/169 | NA | NA | NA |
| Andrisani (2023) | EFTR | 45 | 21.6 ± 8.3 | 25.6 ± 10.6 | 43/45 | 42/45 | 0/45 | 2/45 | 2/45 | 0/45 | NA |
|  | ESD | 45 | 28.7 ± 8.0 | 76.7 ± 26.4 | 42/45 | 36/45 | 3/45 | 1/45 | 1/45 | 3/45 | NA |
| Barbaro (2024) | EFTR | 22 | 21.0 ± 7.4 | 54.0 ± 20.5 | 20/22 | 18/22 | 0/22 | NA | 0/22 | NA | NA |
|  | ESD | 27 | 36.1 ± 8.6 | 61.0 ± 36.2 | 43/27† | 41/27† | 11/27 | NA | 2/27 | NA | NA |

Supplementary Table S2. Clinical outcomes of EFTR versus ESD in included studies

*Binary outcomes reported as Events/Total Patients (e.g., 24/27 = 24 events out of 27 patients).

†Denominator discrepancy in original publication; values reported as published.

EFTR: endoscopic full-thickness resection; ESD: endoscopic submucosal dissection; NA: not available; PPS: post-polypectomy syndrome.

| Study Cohort | Lesion Size (mm) | | LGD Adenoma, n | | HGD Adenoma, n | | SSL, n | | IM-Ca, n | | T1-CRC, n | | SM1 Invasion, n | | SM2/SM3 Invasion, n | | Non-lifting/Fibrotic Lesions, n | | Post-EMR Recurrent Lesions, n | |
| --- | --- | --- | --- | --- | --- | --- | --- | --- | --- | --- | --- | --- | --- | --- | --- | --- | --- | --- | --- | --- |
|  | EFTR | ESD | EFTR | ESD | EFTR | ESD | EFTR | ESD | EFTR | ESD | EFTR | ESD | EFTR | ESD | EFTR | ESD | EFTR | ESD | EFTR | ESD |
| Andrisani 2023 | 21.6 | 28.7 | 25 | 19 | 16 | 26 | 0 | 0 | 0 | 0 | 4 | 6 | 13 | 9 | 5 | 3 | 15 | 15 | 15 | 15 |
| Barbaro 2024 | 17 | 16.7 | 14 | 8 | 0 | 0 | 1 | 5 | 0 | 0 | 5 | 30 | 5 | 30 | 0 | 0 | 20 | 43 | 20 | 43 |
| Bisogni 2020 | 19.3 | 49.1 | 7 | 12 | 7 | 0 | 2 | 0 | 1 | 0 | 4 | 1 | 1 | 0 | 3 | 1 | 8 | 7 | 15 | 6 |
| Falt 2021 | 15.6 | 21.7 | 7 | 8 | 20 | 21 | 0 | 0 | 3 | 5 | 15 | 14 | 10 | 7 | 5 | 7 | 28 | 4 | 28 | 4 |
| Yzet 2023 | 15 | 35 | 79 | 152 | NA | NA | 0 | 0 | NA | NA | 18 | 25 | NA | NA | NA | NA | 98 | 177 | 98 | 177 |

Supplementary Table S3. Summary of Histopathological and Lesion Characteristics

EFTR: Endoscopic full-thickness resection; ESD: Endoscopic submucosal dissection; LGD: low-grade dysplasia adenoma; HGD: high-grade dysplasia adenoma; SSL: sessile serrated lesion; IM-Ca: intramucosal carcinoma; T1-CRC: submucosal invasive colorectal cancer; SM1: submucosal invasion <1000 μm; SM2/SM3: submucosal invasion ≥1000 μm; LST: laterally spreading tumor; NA: not available

Supplementary Table S4. Summary of follow-up characteristics across included studies

| Study Cohort | Median (IQR) follow-up (months) | | Follow-up range (months) | | Loss to follow-up (n) | | Surveillance protocol | Recurrence assessment criteria |
| --- | --- | --- | --- | --- | --- | --- | --- | --- |
|  | EFTR | ESD | EFTR | ESD | EFTR | ESD |  |  |
| Andrisani 2023 | 12 (9–18) | 12 (9–18) | 6–24 | 6–24 | 1 | 0 | Colonoscopy at 6 and 12 months post-procedure; suspicious lesions biopsied for histopathological confirmation | Endoscopic and histologically confirmed residual/recurrent lesion at the resection site |
| Barbaro 2024 | 18 (12–24) | 18 (12–24) | 12–36 | 12–36 | 0 | 0 | Colonoscopy at 3, 6, and 12 months post-procedure, then annually thereafter | Concordant endoscopic and pathological confirmation required |
| Bisogni 2020 | 24 (18–36) | 24 (18–36) | 12–48 | 12–48 | 2 | 1 | Colonoscopy at 1, 3, 6, and 12 months post-procedure, then annually thereafter | Adjudicated by a central panel of 2 independent gastroenterologists |
| Falt 2021 | 18 (12–24) | 18 (12–24) | 6–36 | 6–36 | 1 | 1 | Colonoscopy at 6 and 12 months post-procedure, then annually thereafter | Histopathological confirmation of biopsied suspicious lesions |
| Yzet 2023 | 12 (9–15) | 12 (9–15) | 6–30 | 6–30 | 0 | 0 | Colonoscopy at 6 and 12 months post-procedure | Endoscopic identification of new resection site lesion with histopathological correlation |

IQR: interquartile range;

| Supplementary Table S5. Risk of bias for the included the four non-RCTs, based on the ROBINS-I tool | | | | | | | | | | |
| --- | --- | --- | --- | --- | --- | --- | --- | --- | --- | --- |
| Author | Year | Type of bias | | | | | | | Overall rating | |
|  |  | Confounding | Selection | Measurement classification of interventions | Deviations from intended interventions | Missing data | Measurement of the outcome | Selective reporting of the results |  |  |
| Bisogni | 2020 | Moderate | Moderate | Low | Low | Low | Low | Low | Moderate | |
| Falt | 2021 | Moderate | Moderate | Low | Low | Low | Low | Low | Moderate | |
| Yzet | 2023 | Moderate | Moderate | Low | Low | Low | Low | Low | Moderate | |
| Barbaro | 2024 | Moderate | Moderate | Low | Low | Low | Low | Low | Moderate | |

Supplementary Figure S1 Risk of bias for the included the one RCT, based on the RoB 2 tool.

Supplementary Figure S2. Funnel plots of the resection results of EFTR and ESD. (A) Specimen diameter funnel plot; (B) Procedure time funnel plot; (C) En bloc resection rate funnel plot; (D) R0 resection rate funnel plot.
